# Correlation of effectiveness and tolerability assessments from a pharmacy-based observational study investigating the fixed-dose combination of 400 mg ibuprofen plus 100 mg caffeine for the treatment of acute headache

**DOI:** 10.3389/fneur.2023.1273846

**Published:** 2023-10-24

**Authors:** Charly Gaul, Stefanie Förderreuther, Walter Lehmacher, Thomas Weiser

**Affiliations:** ^1^Headache Center Frankfurt, Frankfurt, Germany; ^2^Department of Neurology, Ludwig Maximilian University, Munich, Germany; ^3^Emeritus, Institute for Medical Statistics, Informatics and Epidemiology, University of Cologne, Cologne, Germany; ^4^Medical Consumer Healthcare, Sanofi, Frankfurt, Germany

**Keywords:** ibuprofen, caffeine, headache, onset of analgesia, epidemiological study

## Abstract

**Introduction:**

Observational studies are valuable for investigating correlations between patient-reported treatment outcomes. In this study, we report a secondary analysis of a published pharmacy-based observational (patient-centered “real-world” outcomes) study on experiences reported by patients who treated their headache with an over-the-counter analgesic.

**Methods:**

A pharmacy-based exploratory survey was conducted in German community pharmacies. Patients buying a fixed-dose analgesic combination product (400 mg ibuprofen + 100 mg caffeine; IbuCaff) to treat their headache were offered a questionnaire that contained—among others—questions about time to onset of pain relief (OPR), assessment of time to onset of pain relief (AOPR), assessment of efficacy and tolerability, and pain intensity 2 h after intake. A correlation analysis of the data was performed. Moreover, perceived treatment effects compared to other acute headache medications used in the past were collected.

**Results:**

The correlation between OPR and AOPR was high (Spearman rank correlation r = 0.594, *p* < 0.0001). Headache patients assessed the onset of analgesic action within 15 min as “very fast” and within 30 min as “fast”. The other readouts were correlated as well [assessment of efficacy and % pain intensity difference (%PID) at 2 h: r = 0.487; OPR/AOPR and %PID at 2 h: r = 0.295/0.318; OPR/AOPR and assessment of tolerability: r = 0.206/0.397; OPR/AOPR and assessment of efficacy: r = 0.406/0.594; assessment of efficacy and assessment of tolerability: r = 0.608; *p* < 0.0001 for all correlations]. Compared to previous treatments, most patients (>89%) assessed the speed of analgesic action, efficacy, and tolerability of IbuCaff as equal to or better than for the previous treatment.

**Discussion:**

Headache patients assessed the onset of analgesia within 15 min as “very fast” and within 30 min as “fast”. Efficacy assessments for acute headache medication appear to be highly correlated.

## 1. Introduction

Acute headache is often treated with over-the counter (OTC) analgesics available in community pharmacies (1). Observational studies, such as pharmacy-based patient surveys, are suited to gain information on patient-reported treatment outcomes and can complement randomized controlled clinical studies, and more adequate observational studies are needed to address aspects that clinical trials cannot explore (2). Such pharmacy-based surveys have been run for cough and cold treatments (3, 4), antispasmodics for gastrointestinal complaints (5, 6), or other acute pain medications (7–9), and their methodology is well established.

Evidence from multiple sources shows that patients expect that acute headache treatments relieve pain as fast and completely as possible. For instance, over 80% of migraine patients ranked complete pain relief and fast onset of action as the most important expectation from their acute pain medication (10). In a questionnaire on patient expectations from migraine treatment, fast pain relief was defined to be important (11). Others used the “willingness to pay” approach and found that patients were willing to pay more money for acute migraine treatment when it acted faster (12). However, the latter two studies did not investigate actual treatments. In contrast, the analysis of two controlled clinical trials investigating sumatriptan for treating acute migraine attacks found that early onset of pain relief correlated with the percentage of pain-free patients (13). How these parameters were correlated with patients' assessment of efficacy was not investigated. Thus, to date, the question of what exactly headache patients mean when they speak of “fast” or “very fast” onset of pain relief is still not clear. Therefore, we analyzed the data from a previously reported, observational, pharmacy-based patient survey (patient-centered “real world” outcomes study) that was run in German community pharmacies and investigated the effects of the fixed-dose combination 400 mg ibuprofen plus 100 mg caffeine (IbuCaff) (9). This product is available without a prescription in Germany and other European countries. The general aim of our analysis was to learn how different efficacy and tolerability readouts were correlated. One focus was how the onset of headache pain relief correlated with the qualitative assessment of speed of action.

## 2. Patients and methods

Details of the survey underlying the present analyses have been reported previously (9). Briefly, patients buying IbuCaff (Thomapyrin TENSION DUO ^®^) between February and June 2019 in German community pharmacies were offered participation in a survey. After giving consent, they were handed a questionnaire that was to be filled out by the patients after taking the product to treat an acute pain event. Questionnaires were sent anonymously in envelopes provided to the participants to the institute that collected the data and performed the primary analysis (Winicker Norimed GmbH, Nuremburg, Germany). The identification of participants was therefore not possible (i.e., full anonymity was provided). No incentive was given to participants. According to German laws and regulations, ethical committee participation was neither required nor recommended for this kind of investigation.

The inclusion criteria were the purchase and use of the respective product, willingness and ability to fill out the questionnaire, usage of the product according to the packaging label, and age of 18 years or older. There were no exclusion criteria. This analysis was restricted to data from patients who reported intake of the product to treat a headache episode. No diagnosis for headache etiology (e.g., tension-type headache or migraine) was performed. The following data on efficacy and tolerability parameters were analyzed: Perceived onset of pain relief (“how long did it take for your pain to start getting better?” OPR; categories: 0–5/6–15/16–30/31–45/46–60/>60 min), assessment of onset of pain relief (“how would you describe the onset of the effect of IbuCaff?”; AOPR; categories: very fast/fast/moderately fast/slow), and assessment of efficacy/tolerability (categories: very good/good/not so good/bad). Pain intensities at baseline and 2 h after intake of the medication (on a 0–10 numerical rating scale) were used to calculate the relative pain intensity difference (%PID). Moreover, participants were asked how they assessed onset of action, efficacy, and tolerability in comparison to the last analgesic product they had taken before to treat a similar pain episode (categories: worse/equal/better).

### 2.1. Data analysis

Data management and primary analysis were done by Winicker Norimed using SAS (version 9.2). Additional analyses were conducted by the authors using GraphPad Prism, version 9.3.1. Spearman's rank coefficients were calculated to assess correlations between OPR and AOPR, OPR and %PID, AOPR and %PID, efficacy ratings and %PID, OPR/AOPR and baseline pain intensity, as well as ratings of efficacy and tolerability. Relative and cumulative relative frequencies of OPR categories stratified by AOPR categories were calculated; rate ratios (relative rates) of these cumulated relative frequencies were used to assess which AOPR category steps discriminated between cumulative OPR rates. The relative rate (RR) cutoff for the discrimination between AOPR categories was set to a value of 2.

## 3. Results

**Figure 1:**
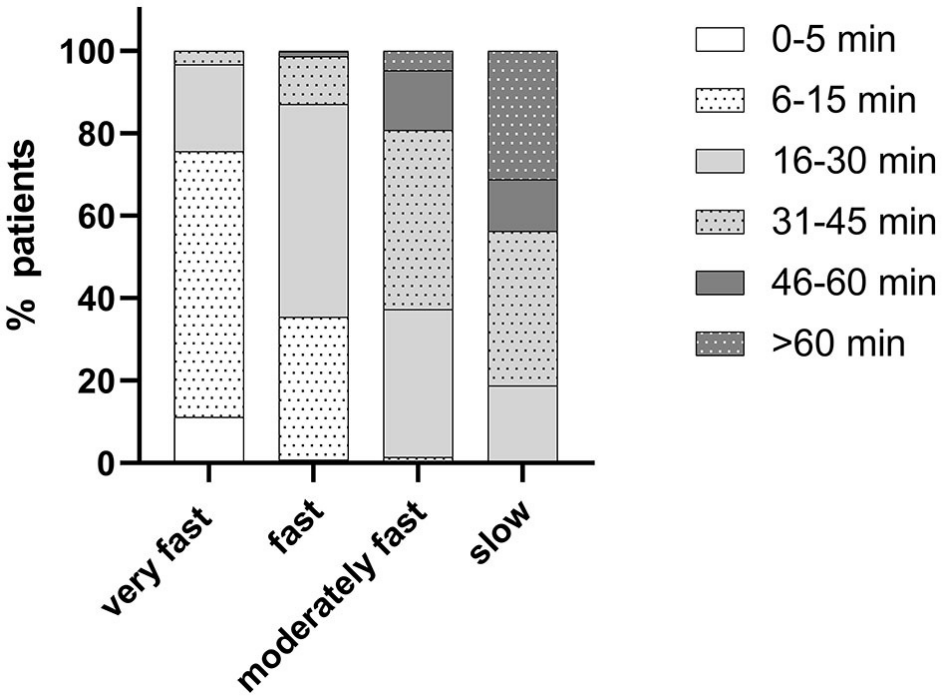
Onset of pain relief (OPR) data stratified by the assessment of onset of pain relief (AOPR) categories.

In total, 1,124 participants provided analyzable questionnaires, 229 treated other pain than headache, and 895 of them reported the use of IbuCaff for treating a headache episode (9). The correlation between OPR and AOPR was high (Spearman r = 0.594; 95% CI: 0.5468 to 0.6373; *p* < 0.0001). Most of the participants experienced OPR as “very fast” (21.8%) or “fast” (58.8%), and only 17.5% and 1.9% assessed it as “moderately fast” or “slow,” respectively (Supplementary Table). Figure 1 gives a graphical representation of the OPR data stratified by the four AOPR categories. A total of 76% of patients reporting onset of pain relief within 15 min assessed this as “very fast,” and 87% of those reporting onset within 30 min assessed this as “fast”.

Next, we used the cumulated relative frequencies to assess which AOPR category steps discriminated between cumulative OPR rates. Comparison of RRs for the different OPR values for AOPR categories “very fast” vs. “fast” showed that these rates were relatively high for OPR ≤ 5 min, >2 for OPR ≤ 15 min, and then leveled around a value of 1. Thus, OPR later than 15 min made no difference for the two AOPR categories and, in consequence, suggests that this is the cutoff point for the distinction between AOPR of “very fast” vs. “fast”. For the AOPR category “fast” vs. “moderately fast,” this cutoff OPR was 30 min. The analysis of the AOPR category “moderately fast” vs. “slow” gave no interpretable results (Table 1).

**Table 1:** Ratios of cumulative relative frequencies of OPR categories (RR) with 95% confidence intervals (CI) and *p*-values for the comparisons of different assessments of onset of pain relief.

	**“very fast” vs. “fast”**	**“fast” vs. “moderately fast”**	**“moderately fast” vs. “slow”**
	**[RR (CI)** ***p*****]**	**[RR (CI)** ***p*****]**	**[RR (CI) p]**
≤ 5 min	13.5 (4.67;38.95) *p* < 0.0001	Not defined	Not defined
≤ 15 min	2.13 (1.84;2.47) *p* < 0.0001	25.65 (6.44;102.15) *p* < 0.0001	Not defined
≤ 30 min	1.11 (1.06;1.16) *p* < 0.0001	2.33 (1.88;2.89) *p* < 0.0001	1.98 (0.70;5.63) *p* = 0.196
≤ 45 min	1.01 (1.004;1.02) *p* = 0.0082	1.22 (1.12;1.32) *p* < 0.0001	1.43 (0.92;2.22) *p* = 0.1075
≤ 60 min	1.002 (0.99;1.006) *p* = 0.3173	1.05 (1.01;109) *p* = 0.0117	1.12 (0.89;1.42) *p* = 0.3261
>60 min	1 (1.00;1.00) *p* = 1	1 (1.00;1.00) *p* = 1	1 (1.00;1.00) *p* = 1

**Figure 2:**
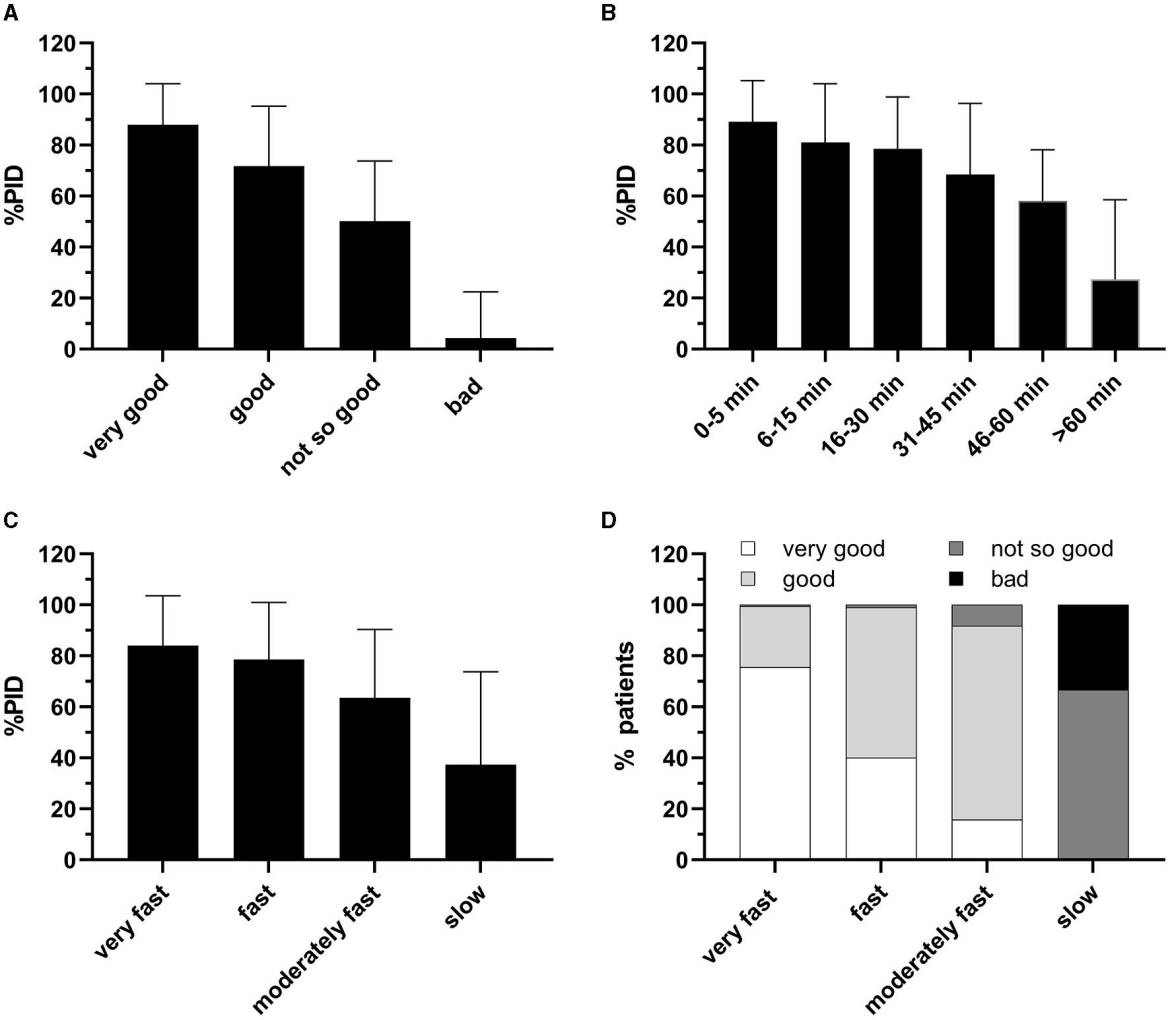
**(A)** %PID at 2 h by assessment of efficacy; **(B)** % PID at 2 h by onset of pain relief (OPR); **(C)** %PID at 2 h by assessment of time to onset of pain relief (AOPR). **(D)** Assessment of tolerability by AOPR. Data in **(A–C)** are shown as means with standard deviations.

Figure 2 shows graphical representations of other correlations. A higher %PID at 2 h corresponded to higher percentages of patients with higher ranking efficacy (Figure 2A), earlier OPR (Figure 2B), and AOPR (Figure 2C). AOPR was also correlated with the assessment of tolerability (Figure 2D). Baseline pain intensity correlated only weakly with OPR but not AOPR. OPR and AOPR correlated with efficacy and tolerability outcomes, and perceived efficacy was correlated with tolerability as well. More detailed information on correlation analysis of the various parameters is given in Table 2.

**Table 2:** Correlations of analyzed parameters.

**Variable 1**	**Variable 2**	**Spearman r**	**95% CI**	***P*-value**
OPR	Baseline PI	0.1186	0.0489 to 0.1871	0.0006
AOPR	Baseline PI	0.0500	−0.0201 to 0.1198	0.0750
OPR	% PID 2 h	0.2947	0.2293 to 0.3573	< 0.0001
AOPR	% PID 2 h	0.3178	0.2533 to 0.3795	< 0.0001
OPR	Assessment efficacy	0.4064	0.3464 to 0.4630	< 0.0001
AOPR	Assessment efficacy	0.5936	0.5464 to 0.6371	< 0.0001
Assessment of efficacy	% PID 2 h	0.4869	0.4316 to 0.5387	< 0.0001
OPR	Assessment tolerability	0.2065	0.1387 to 0.2723	< 0.0001
AOPR	Assessment tolerability	0.3968	0.3363 to 0.4540	< 0.0001
Assessment efficacy	Assessment tolerability	0.6079	0.5620 to 0.6501	< 0.0001

**Figure 3:**
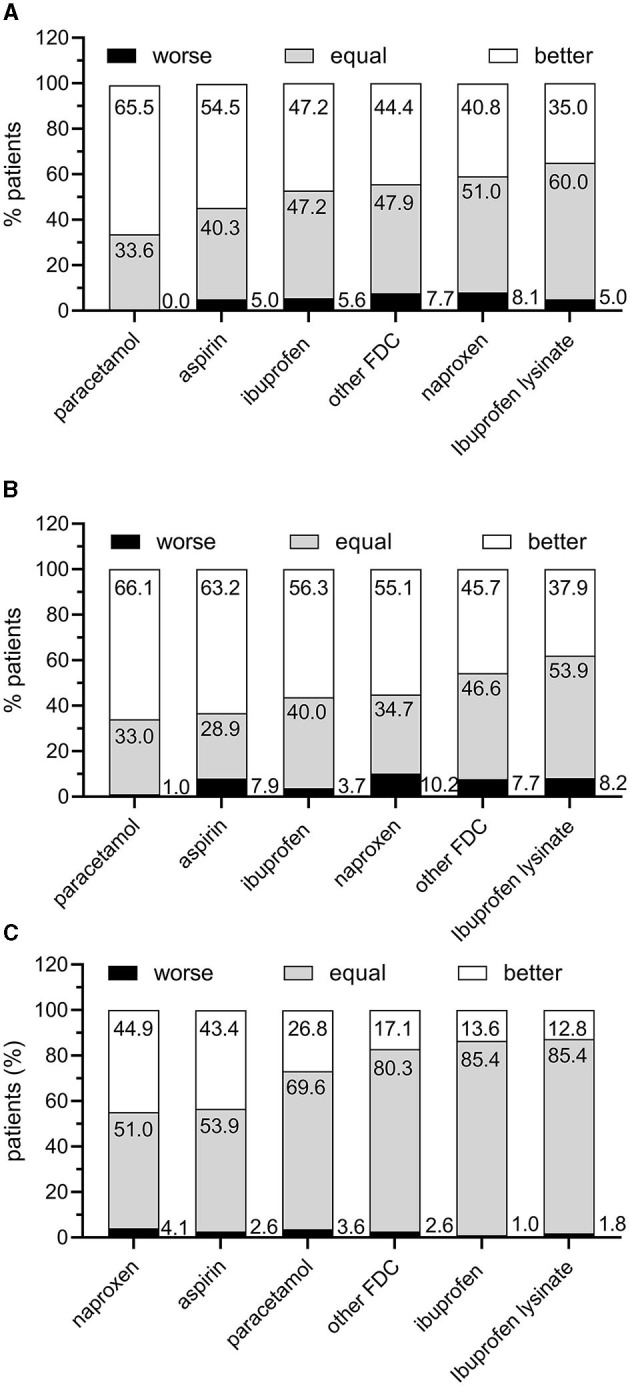
Patient perception of IbuCaff compared to the last acute medication taken to treat a similar pain episode. **(A)** Assessment of speed of analgesic action. **(B)** Assessment of efficacy. **(C)** Assessments of tolerability.

Participants were asked which other acute treatment they had taken before IbuCaff for the last comparable pain episode, and how they assessed overall efficacy, how fast the analgesic acted, and overall tolerability in comparison to IbuCaff. Relative percentages of assessments are shown in Figure 3. Only low percentages of participants rated efficacy worse compared to the last individual treatment (between 0% and 8.1%; Figure 3A). The percentages of those who ranked IbuCaff efficacy better ranged from 35% (in comparison to ibuprofen lysinate) to 65.5% (in comparison to paracetamol). Between 1.0% (paracetamol) and 10.2% (naproxen) reported that IbuCaff acted not as fast as the previously taken analgesic, and between 37.5% (ibuprofen lysinate) and 66.1% (paracetamol) found IbuCaff to act faster (Figure 3B). The tolerability of IbuCaff was rated lower by between 1.0% (ibuprofen) and 4.1% (naproxen) and higher by 12.8% (ibuprofen lysinate) to 44.9% (naproxen; Figure 3C).

## 4. Discussion

Pharmacy-based patient surveys can gain information on how patient-reported readouts are correlated and complement data obtained from randomized controlled clinical trials (2). One limitation of studies with this design is that they do not allow for the comparison of an active treatment with a control (e.g., placebo) group and, therefore, cannot be used to show, e.g., efficacy in general. Since the combination of ibuprofen and caffeine (400/200 mg) has been shown to be superior compared to 400 mg ibuprofen in tension-type headache (14) and meta-analyses of controlled trials on ibuprofen for treating tension-type and migraine headaches and for the effects of caffeine as co-analgesic are available (15–17), it is beyond doubt that IbuCaff can reduce acute headache. Other limitations are that no headache diagnosis was performed and that for assessing historical treatment, data recall bias could not be excluded. One strength of this study design is the focus on the patient, and studies of this kind allow for characterizing patient demographics and behaviors, as well as perceived treatment effects in general practice (3–9). Another strength of this study is that it allows for more general conclusions of the kind “if a headache treatment has an effect X, how does this correlate with effect Y,” which can be assumed to be independent of the treatment *per se* and therefore should be transferrable to any abortive headache treatment.

Our study shows that the onset of headache pain relief earlier than 15 min after intake of IbuCaff was perceived as “very fast” by the patients and onset earlier than 30 min was perceived as “fast”. Altogether more than 80% of patients belonged to these two categories. The onset of pain relief within the first 5 min after intake was reported by 24 patients and most probably is due to placebo effects because even under best conditions (i.e., in the fasted state), only a small amount of ibuprofen and caffeine from IbuCaff can be expected to be absorbed that fast (18). A randomized controlled trial on dental extraction pain showed that IbuCaff was superior to 400 mg ibuprofen as early as 15 min after intake (19) and, therefore, the reported onset of pain relief in the category (“6–15 min” and later) can be assumed to reflect a pharmacological effect.

Interestingly, other patient-reported efficacy parameters were correlated with OPR and AOPR as well. The earlier the pain was relieved, the higher the %PID was compared to baseline at 2 h. This is not surprising if one assumes a steady increase in pain relief over the time up to 2 h after intake of an analgesic. In consequence, an early onset of pain relief was correlated with the overall impression of treatment efficacy. Correlations of baseline pain with OPR and AOPR were very weak to absent.

The strong correlation between the assessment of efficacy of an analgesic and %PID has been shown in a randomized controlled trial before (20).

Somewhat surprising was the correlation between AOPR and assessment of tolerability: Early pain relief and high tolerability went hand in hand. Controlled clinical trial occurrence of untoward effects was correlated with treatment efficacy/pain reduction [i.e., lower tolerability was correlated with higher efficacy; (21–23)]. One explanation might be that adverse events lead to unblinding in a controlled clinical trial (since patients experiencing adverse events will assume that they have received an active treatment), which cannot be the case in our study. Further studies will be needed to explain this finding.

One guideline-recommended endpoint for clinical trials investigating migraine headache is the analysis of treatment preference (in comparison to prior treatments) (24). Although in our study no formal diagnosis for the headache etiology was performed, it can be hypothesized that various migraine-trial relevant parameters can be transferred to acute headache in general.

In terms of perceived efficacy, onset of action, and tolerability, IbuCaff was rated worse than previous OTC treatments by only small percentages of patients (usually in the one-digit range). On the other hand, large percentages of patients rated IbuCaff better than the comparators. This is not surprising since caffeine-containing analgesics have been shown to be superior to those without caffeine (16). In comparison to other analgesics (including ibuprofen and ibuprofen lysinate), tolerability was generally rated at least as equal.

Comparisons with historical data bear the risk of bias (such as recall bias). If this was the case for our analysis, one should expect random distributions, which was obviously not the case. For the parameters such as “perceived efficacy” and “onset of effect,” the highest percentages reporting superiority were found for paracetamol [a relatively weak analgesic; (25)] and the lowest for ibuprofen lysinate. For “tolerability,” the highest superiority was reported compared to naproxen and lowest for ibuprofen lysinate. Thus, the assessments are not randomly distributed, which underpins their validity. Moreover, probably not all patients have found their optimal acute headache treatment yet, and a different analgesic might work better for them. In consequence, each patient should be encouraged to try various acute pain analgesics to find the one that works the best individually.

Taken together, this analysis shows that an abortive headache treatment relieving pain within 15 min after intake can be considered to act “very fast” from the patient's perspective and that within 30 min can be considered to act “fast”. The fast onset of analgesic effects was positively correlated with several other efficacy readouts, suggesting that an early onset of action is a key factor for successfully treating acute headache. This might be an interesting aspect of patient education: when pain relief starts early, chances are high for an overall successful headache treatment.

## Data availability statement

Publicly available datasets were analyzed in this study. Qualified researchers may request access to data and related study documents including the study report, study protocol with any amendments, statistical analysis plan, and dataset specifications. Further details on Sanofi's data sharing criteria, eligible studies, and process for requesting access can be found at: https://www.vivli.org/.

## Ethics statement

The manuscript represents a secondary analysis of a published study. Ethical review and approval was not required for the study on human participants in accordance with the local legislation and institutional requirements. The patients/participants provided their written informed consent to participate in this study.

## Author contributions

CG: Conceptualization, Investigation, Writing—review and editing. SF: Conceptualization, Investigation, Writing—review and editing. WL: Conceptualization, Investigation, Methodology, Writing—review and editing. TW: Conceptualization, Investigation, Methodology, Project administration, Writing—original draft.
