# Supplementary material for: Correlation of effectiveness and tolerability assessments from a pharmacy-based observational study investigating the fixed-dose combination of 400 mg ibuprofen plus 100 mg caffeine for the treatment of acute headache
Source: Front Neurol. 2023 Oct 24;14:1273846. doi: 10.3389/fneur.2023.1273846 (PMC10628638; doi:10.3389/fneur.2023.1273846)
Supplement: Supplementary file 1 [file Data_Sheet_1.PDF]

Supplementary Material

1 Supplementary Table

Frequencies of patients reporting assessments of AOPR and OPR.

	very fast	fast	moderately fast	slow	sum
0-5 min	20	4	0	0	24
6-15 min	116	168	2	0	286
16-30 min	38	251	52	3	344
31-45 min	6	56	63	6	131
46-60 min	0	6	21	2	29
>60 min	0	1	7	5	13
sum	180	486	145	16	827
